# HIV Capsid Inhibitors Beyond PF74

**DOI:** 10.3390/diseases7040056

**Published:** 2019-10-30

**Authors:** Carole McArthur, Fabio Gallazzi, Thomas P. Quinn, Kamal Singh

**Affiliations:** 1 Department of Oral and Craniofacial Sciences, School of Dentistry, University of Missouri, Kansas City, MO 64108, USA; 2 Kansas City School of Medicine, University of Missouri, Kansas City, MO 64108, USA; 3 Department of Pathology, Truman Medical Center, Kansas City, MO, 64108, USA; 4 Bond Life Sciences Center, University of Missouri, Columbia, MO 65211, USA; gallazzif@missouri.edu; 5 Department of Chemistry, University of Missouri, Columbia, MO 65211, USA; 6 Department of Biochemistry, University of Missouri, Columbia, MO 65211, USA; QuinnT@missouri.edu; 7 Department of Molecular Microbiology and Immunology, University of Missouri, Columbia, MO 65211, USA; 8 Division of Clinical Microbiology, Department of Laboratory Medicine, Karolinska Institute, 14186 Stockholm, Sweden

**Keywords:** human immunodeficiency virus, capsid, assembly, small molecule inhibitors, PF74, GS-CA1, GS-6207, disassembly, uncoating

## Abstract

Human immunodeficiency virus (HIV) capsid plays important roles at multiple stages of viral replication. At the initial stages, controlled uncoating (disassembly) of the capsid ensures efficient reverse transcription of the single-stranded RNA genome, into the double-stranded DNA. Whereas at later stages, a proper assembly of capsid ensures the formation of a mature infectious virus particle. Hence, the inhibition of capsid assembly and/or disassembly has been recognized as a potential therapeutic strategy, and several capsid inhibitors have been reported. Of these, PF-3450074 (PF74) has been extensively studied. Recently reported GS-CA inhibitors (GS-CA1 and GS-6207), have shown a strong potential and appear to contain a PF74 scaffold. The location of resistance mutations and the results of structural studies further suggest that GS-CA compounds and PF74 share the same binding pocket, which is located between capsid monomers. Additionally, phenylalanine derivatives containing the PF74 scaffold show slightly enhanced capsid inhibiting activity. A comparison of capsid structures in complex with host factors and PF74, reveals the presence of common chemical entities at topologically equivalent positions. Here we present the status of capsid inhibitors that contain PF74 scaffolds and propose that the PF74 scaffold may be used to develop strong and safe capsid inhibitors.

## 1. Introduction

Currently recommended antiretroviral therapies (cART) effectively suppress HIV and maintain HIV viral load below the detection levels (<50 copies/mL). As a result, HIV/AIDS is now a chronic and manageable disease [1,2,3,4,5,6]. However, HIV diversity, HIV persistence and the emergence of or the transfer of drug resistance mutations (DRMs) challenges the optimal outcome. Hence, new antiviral targets and agents that can inhibit HIV-1 replication through novel mechanisms are constantly sought. One such target is the HIV capsid, a structural protein that plays important roles at multiple stages of viral replication.

**Figure 1:**
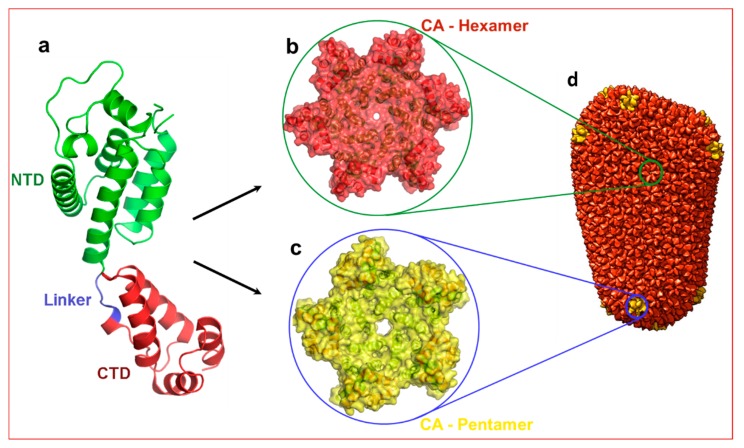
Structure of HIV-1 capsid. (**a**) Structure of capsid (CA) monomer. This figure was generated from the X-ray crystal structure of native HIV-1 capsid protein bound to PF74 [9] (PDB entry 4XFZ). NTD: N-terminal domain. CTD: C-terminal domain. Panels (**b**,**c**) show hexamer and pentamers formed from the same CA monomer. (**d**) Approximately 250 hexamers and 12 pentamers form a conical shaped capsid that houses necessary and sufficient components to initiate reverse transcription immediately after infection. Panel d is reproduced from PDB101 with permission.

The capsid monomer (CA) is a 24 kDa α-helical protein with two distinct domains: an N-terminal domain (NTD) and a C-terminal domain (CTD) connected by a five residue flexible linker (Figure 1). During late stages of viral replication, the Gag polyprotein localizes at the plasma membrane and buds off as spherical, immature non-infectious virus. Auto-cleavage of the viral protease during maturation and the subsequent processing of the Gag polyprotein generates several structural proteins and small peptides, including capsid monomers (Figure 1a). These CA monomers then assemble into ~250 hexamers (Figure 1b) and 12 pentamers (Figure 1c) to form a mature fullerene-like core structure known as capsid core (hereafter referred to as capsid) (Figure 1d) [7,8].

At the early stages of HIV-1 infection, i.e., after infection and the fusion of the viral envelope with the host cell membrane, capsid is released into the cytosol where it undergoes controlled disassembly (uncoating). While the timing, mechanism, and extent of capsid uncoating is controversial [10,11,12,13,14,15,16,17,18], published reports suggest that capsid uncoating is associated with the initiation or continuation of the reverse transcription [14,19,20,21,22,23,24], and integration of viral DNA into the host chromosome [17,20,25,26,27,28]. Cosnefroy et al. [29] showed that HIV-1 capsid uncoating initiates after the first strand transfer of reverse transcription. Another report demonstrated that HIV-1 core undergoes morphological changes during reverse transcription [30]. These studies suggest that reverse transcription initiates within the virus particle and continues with capsid disassembly in the cytoplasm of the infected cell. The uncoating of capsid also involves the interaction of capsid with viral proteins (e.g., reverse transcriptase and integrase) [12,13], and host proteins including cyclophilin A [17], kinesin [31], dynein [31,32], microtubules [33], nucleoporins NUP358 and NUP153 [34], cleavage and polyadenylation specificity factor 6 (CPSF6) [35,36], transportin 3 (TNPO3) [34], and β-karyopherin Transportin-1 (TRN-1) [37]. The uncoating of capsid is influenced by certain mutations that change stability. Second, interactions between CA protomers alter replication events that may severely affect viral infectivity [21,38]. The role of capsid in the above-mentioned processes highlights capsid as an attractive antiviral target for the development of inhibitors [39,40,41].

## 2. PF74 and Capsid Inhibitors Containing the PF74-Scaffold

**Figure 2:**
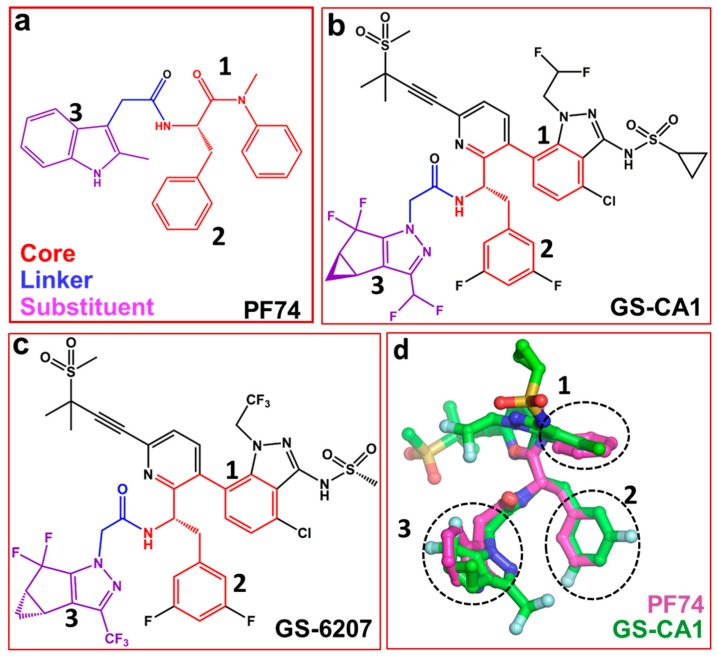
Structures of PF74 and GS-CA compounds. (**a**) PF74 can be divided into three components: polyphenyl core (red), linker (blue) and substituent (violet). Numbers 1, 2 and 3 represent three ring structures in PF74. Panels (**b**) and (**c**) show the chemical structure of GS-CA1 and GS-6207 compounds, respectively. Chemical moieties colored red, blue and violet correspond to those in PF74 (panel a). (**d**) Superposition of PF74 and GS-CA1. Dotted circles show the superposition of three ring structures present in PF74, GS-CA1 and GS-6207.

Several capsid-binding small molecules, which inhibit viral replication have been identified [41,42,43,44,45,46,47,48,49,50,51,52]. One of the most studied inhibitors is PF-3450074, also known as PF74 (Figure 2a) [25,53,54,55]. It inhibits infection, blocks reverse transcription [56] and prevents replication in vitro [3]. PF74 binds in a pocket between the NTD and CTD of monomers [45,57,58]. The structure of PF74 can be divided in to three components: a polyphenyl core (red, Figure 2a), a linker (blue, Figure 2a) and an indole substituent (violet, Figure 2a) [59]. In the following sections, we discuss compounds that contain PF74 scaffolds and have potential as future capsid inhibitors.

Recently reported two capsid inhibitors, GS-CA1 (Figure 2b) and GS-62072 (an analog of GS-CA1) (Figure 2c) display greater potency than currently approved antivirals in vitro [60,61]. GS-CA1 inhibits HIV-1 replication in T-lymphocytes and peripheral blood mononuclear cells (PBMCs) with EC_50_s of 240 pM and 140 pM, respectively) [60]. Alternatively, GS-6207 inhibits HIV-1 in MT-4 cells and PBMCs with EC_50_s of 100 pM and 50 pM, respectively [61]. Both compounds also have a potential as long acting inhibitors [60,62,63]. Analyses of the structures of GS-CA1 and GS-6207 show that both compounds contain a polyphenyl core and the linker region similar to that in PF74 (Figure 2a–c).

The structure of GS-CA1/CA complex has been solved, but it is not available in the public domains such as the Protein Data Bank [64]. However, molecular docking studies shows that both GS-CA1, GS-6207and PF74, share the same binding site [65] which is located between capsid NTD and CTD [57,60,66]. The docking results show the rings of PF74 superpose on the different rings of GS-CA1 (dotted circles 1, 2, and 3 in Figure 2d). More specifically, one of the two phenyl rings of PF74 is at a topologically equivalent position in the indazole ring of GS-CA1 (Figure 2d, circle 1), and the other phenyl ring of PF74 superposes on the difluorobenzene ring of GS-CA1 (Figure 2d, circle 2). The indole ring of PF74 superposes closely on the tetrahydrocyclopenta-pyrazole ring of GS-CA1 (Figure 2d, circle 3). In addition, the polar groups of the two molecules (PF74 and GS-CA1) superpose well [65].

In vitro resistance selection experiments have identified mutations L56I, M66I, Q67H, N74D and A105E under GS-CA1 pressure [67]. Mutations Q67H, N74D, K70N, Q67H/N74S, Q67H/T107N, L56I, Q67H/N74D, M66I and T107N are associated with GS-6207 resistance in vitro [68]. Several of these mutations are either associated with PF74 resistance or they are in the vicinity of PF74 binding pocket [45,55,56,58,69]. These data further support molecular docking studies and implicate that GS-CA1 binds at the PF74 binding pocket as predicted recently [65].

Together with common structural moieties in GS-CA1, GS-6207 and PF74, the GS-CA compounds have additional chemical groups that have extensive interactions with the NTD of adjacent capsid monomer. More specifically, methanesulfonyl moiety of GS-CA1 and GS-6207 is within interacting distance of I37, P38, S41, I135 and N139 [65]. It is possible that these interactions result in a better binding affinity of CA with GS-CA1 than PF74 [65], which results in the enhanced potency of GS-CA compounds.

**Figure 3:**
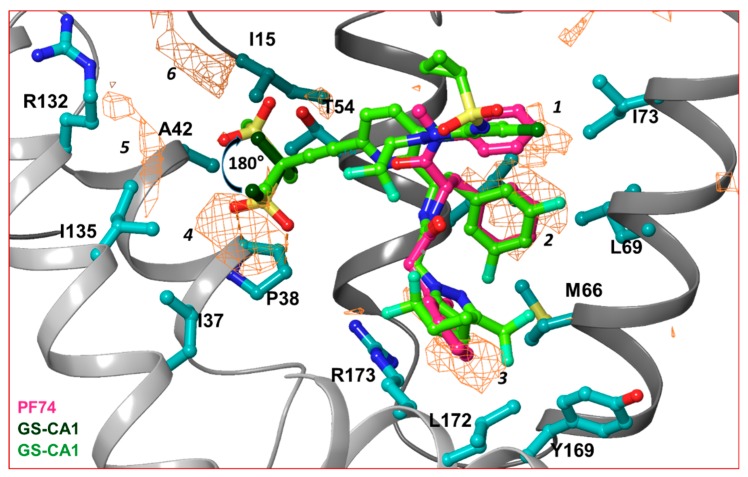
Hydrophobic pockets between two capsid monomers. This figure shows the hydrophobic pockets (orange wire-mesh) present between two capsid monomers of a hexamer. The amino acid residues that contribute in formation of hydrophobic pockets are displayed in ball-and-stick representation. The presence of hydrophobic pockets 4, 5 and 6 suggests that the conformation of methanesulfonyl moieties of GS-CA1 and GS-6207 can assume a conformation that is 180° rotated from previously predicted [65]. Note that hydrophobic pockets 1, 2 and 3 are at a topologically equivalent position to three ring structures of PF74, GS-CA1 or GS-6207.

In order to dissect the small molecule binding site between two capsid monomers of an hexamer, we employed the SiteMap program [70] of Schrödinger Suite (Schrödinger Inc., NY) in the crystal structure of the hexamer/PF74 complex (after removing PF74) (PDB file 5HGL [69]). Six hydrophobic pockets between two capsid monomers, as predicted by the SiteMap program, are shown as orange wire-mesh in Figure 3. Three of these hydrophobic pockets (marked 1, 2 and 3 in Figure 3) are present at the topologically equivalent positions of the three ring-structures of PF74/GS-CA compounds; regions 4, 5 and 6 are close to methanesulfonyl moieties of GS-CA compounds (Figure 3). This analysis suggests that methanesulfonyl moiety of GS-CA compounds may adopt a conformation that is rotated by ~180° from the one predicted recently [65]. This analysis also indicates that the methyl groups of methanesulfonyl moiety may have been strategically incorporated in GS-CA compounds to improve binding to the capsid. It is unclear if methanesulfonyl groups contribute to the low solubility of these compounds. In spite of low solubility [61] and violation of theoretical rules of drug-likeliness [61,71,72], GS-CA compounds have a strong potential to be developed as long-acting drugs.

The proposal that interactions of compounds with both capsid monomers involved in binding site formation may enhance potency of PF74-based molecules, was recently studied by synthesizing phenylalanine derivatives and testing their inhibitory activity [59]. One of these compounds had a selectivity index (SI) (CC_50_/EC_50_) of 13.33 compared to the SI of PF74 (SI = 11.85) [59]. These results suggest that the PF74 scaffold can be used to develop better capsid inhibitors.

Another reported capsid inhibitor BI-2 that contains a partial PF-74 polyphenyl core binds at the PF74 binding site [57]. A superposition of capsid bound to PF74 (PDB files 4XFZ and 5HGL) [9,69] and BI-2 (PDB file 4U0F) [57] demonstrates that two phenyl rings of BI-2 superposed well with the phenyl rings of PF74 (Figure 2b) [65]. BI-2 does not have a chemical moiety equivalent to the indole ring of PF74, which forms some contacts with CTD. It appears that a greater potency of PF74 compared to BI-2 is also the result of interactions of the indole ring with capsid.

## 3. Similarity among PF74, PF74-Based Compounds and Host Factors

CPSF6 and NUP153 are host factors that have been implicated in nuclear entry of HIV-1 through their interactions with capsid [66,73,74,75,76,77,78,79]. CPSF6 is a pre-mRNA splicing factor and member of the serine/arginine-rich (SR) protein family [80]. It is predominately a nuclear protein but localizes with capsid in the cytoplasm [25,81]. Depletion of CPSF6 marginally increases viral infection [73,81]. NUP153 is a nucleoprotein located at the nucleus side of the nuclear pore complex (NPC) [74]. NUP153 participates in HIV-1 translocation to nucleus through the nuclear pore [16,76]. The role of CPSF6 and NUP153 together with other host factors in HIV-1 infection have been reviewed by Yamashita and Engelman [81].

**Figure 4:**
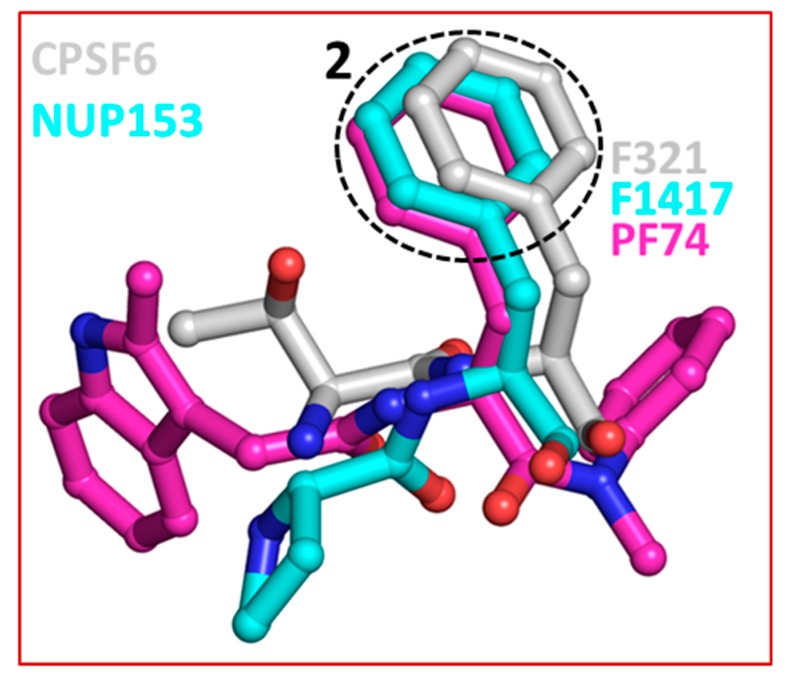
Superposition of CPSF6, NUP153 and PF74. The dotted circle highlights the equivalent position of phenylalanine residues in CPSF6 and NUP153 and one of the two phenyl rings of PF74. The equivalent ring structure in GS-CA1 and GS-6207 is difluorobenzene, which is not shown in this figure. The structures of capsid inhibitors shown here have either been solved or in complex with capsid.

The crystal structures of capsid bound to the peptides from CPSF6 (313–327) and NUP153 (1407–1423) have been reported [57]. Structural comparison shows that both CPSF6 and NUP153 peptides bind at the PF74/BI-2 binding site [52,57]. Therefore, as expected both PF74 and BI-2 interfere with CPSF6 or NUP153 binding to the capsid [57,66]. This comparison also shows that F321 of CPSF6 and F1417 of NUP153 perfectly superpose on the phenyl ring of PF74 (or BI-2) (Figure 4) [65]. As discussed above (Section 2), two most potent capsid inhibitors GS-CA1 and GS-6207 also contain a similar structural moiety (difluorobenzyl) at the topologically equivalent position. This analysis further suggests that chemical features present in host factors can be exploited to develop capsid inhibitors. Indeed, a cyclic peptide (Pep-1) containing chemical features of CPSF6, NUP153, PF74 and BI-2, bind capsid hexamers with nanomolar affinity [65]. Common structural moieties at topologically equivalent positions in CPSF6, NUP154, PF74, BI-2, GS-CA1, GS-6207 and Pep-1 [65] are shown in Table 1.

## 4. Capsid Inhibitors without PF74 Scaffolds

Other reported capsid inhibitors include benzodiazepine (BD) and benzimidazole (BM) compounds [82,83,84], CAP-1 [43,46], or peptide inhibitors, such as NYAD-1. These compounds do not bind at the PF74 binding site and they inhibit capsid and mature virus by different mechanisms. For example, NYAD-1 disrupts the formation of both immature- and mature-like virus particles [85] and it inhibits viral replication [44,49,52].

## 5. Conclusions

In conclusion, here we present the current predicted mechanisms of capsid inhibitors containing PF74 scaffold. PF74, although used extensively in experimental settings, has poor metabolic properties suggesting that improved capsid inhibitors can be developed using PF74 as the starting point.

## Figures and Tables

**Table 1:** Structural similarity among PF74, PF74-based compounds and host factors.

PF74	BI-2	CPSF6	NUP153	GS-CA1	GS-6207	Pep-1
phenyl	phenyl	F321	F1417	difluorobenzyl	difluorobenzyl	phenylalanine
phenyl	phenol	-	-	Indazole	Indazole	proline
indole	-	G318-Q319^1^	-	cyclopenta-pyrazole	cyclopenta-pyrazole	valine
-	-		F1415	methanesulfonyl	methanesulfonyl	phenylalanine

^1^ A part of G318-Q319 dipeptide of CPSF6 is topologically close to the indole ring of PF74.
